# Dreaming during the Covid-19 pandemic: Computational assessment of dream reports reveals mental suffering related to fear of contagion

**DOI:** 10.1371/journal.pone.0242903

**Published:** 2020-11-30

**Authors:** Natália Bezerra Mota, Janaina Weissheimer, Marina Ribeiro, Mizziara de Paiva, Juliana Avilla-Souza, Gabriela Simabucuru, Monica Frias Chaves, Lucas Cecchi, Jaime Cirne, Guillermo Cecchi, Cilene Rodrigues, Mauro Copelli, Sidarta Ribeiro

**Affiliations:** 1 Brain Institute, Federal University of Rio Grande do Norte – UFRN, Natal, Brazil; 2 Department of Physics, Federal University of Pernambuco – UFPE, Recife, Brazil; 3 Department of Modern Foreign Languages and Literature, Federal University of Rio Grande do Norte – UFRN, Natal, Brazil; 4 Department of Linguistics, Pontifícia Universidade Católica do Rio de Janeiro – PUC RJ, Rio de Janeiro, Brazil; 5 The Pennsylvania State University, University Park, Pennsylvania, United States of America; 6 IBM T.J. Watson Research Center, Yorktown Heights, New York, United States of America; IRCCS Fondazione Santa Lucia, ITALY

## Abstract

The current global threat brought on by the Covid-19 pandemic has led to widespread social isolation, posing new challenges in dealing with metal suffering related to social distancing, and in quickly learning new social habits intended to prevent contagion. Neuroscience and psychology agree that dreaming helps people to cope with negative emotions and to learn from experience, but can dreaming effectively reveal mental suffering and changes in social behavior? To address this question, we applied natural language processing tools to study 239 dream reports by 67 individuals, made either before the Covid-19 outbreak or during the months of March and April, 2020, when lockdown was imposed in Brazil following the WHO’s declaration of the pandemic. Pandemic dreams showed a higher proportion of anger and sadness words, and higher average semantic similarities to the terms “contamination” and “cleanness”. These features seem to be associated with mental suffering linked to social isolation, as they explained 40% of the variance in the PANSS negative subscale related to socialization (p = 0.0088). These results corroborate the hypothesis that pandemic dreams reflect mental suffering, fear of contagion, and important changes in daily habits that directly impact socialization.

## Introduction

Since the dawn of civilization, the relationship between dreams and fear has long been expressed across different cultures [1]. Where ancient and some contemporary cultures interpret dreams as divine advice, neuroscience postulates a memory mechanism that metabolizes negative emotions [2] and simulates threats to prepare individuals for the challenges of tomorrow [3] and allows them to learn from experience [4]. Recurrent dreams depicting threatening situations have proven to be an important sign in the diagnosis of post-traumatic stress disorder [5–11]. The ongoing Covid-19 pandemic, as a global threat of deadly contagion, required a sudden adaptation to unprecedented levels of social isolation. Quarantines and lockdowns affect people’s lives in different ways, from the reassessment of their economic perspectives to the effort they must make to maintain or establish safe and meaningful relationships. Do dreams reflect the planetary crisis and our urgent need to adapt? Do dreams express our inner suffering? Can they mitigate the challenges we face, and generate solutions?

There has been intense academic debate about the function of dreams and their underlying brain mechanisms [12, 13]. The development of the concept of “day-residue” in psychoanalytic theory and therapy [14] showed that events experienced during the previous day can be identified if dreams are carefully scrutinized. While the prevalence of episodic memories (specific memories of lived experiences) in dream reports is a matter of debate [5, 15–20], it is undeniable that waking life is the main source of oneiric images, and recent studies have shown that spatial and temporal fragments of episodic memories are present in dream reports [5, 6, 12]. Among the several divergent theories about the role of dreams as a virtual simulation of waking life, three stand out as potentially relevant for interpreting how the Covid-19 pandemic might affect dreaming: 1) the Threat Simulation Theory (TST) [3], 2) the Emotion Regulation Theory (ERT) [2], and 3) the Social Simulation Theory (SST) [4].

The Threat Simulation Theory [3] postulates that the retrieval of threatening memories during sleep gave our ancestors an evolutionary advantage in coping with the challenges of waking reality. According to TST, dreaming evolved as a mechanism to train and learn new survival strategies in the protected environment of offline mental experience. The Emotion Regulation Theory posits that the function of dreams is to regulate emotions through psychological and neural mechanisms such as desomatization with contextualization, leading to the resolution of emotional problems and to the extinction of fear memories [2]. According to ERT, as a virtual reality free of physical danger, dreaming increases the exposure to emotionally negative situations, eventually leading to memory reconsolidation with decreased emotional load [2], while nightmares can be understood as an extreme situation that pushes this physiological mechanism to its limits [2]. Finally, the Social Stimulation Theory sees daily memory retrieval as a useful mechanism for training new social-behavioral strategies that can be tested in a safe environment, free of the social consequences of potentially bad choices [4]. These three theories converge towards the assumption that when people are faced with a challenging, threatening and socially disruptive situation, daytime memories having emotional impact are somehow processed during dreams.

The ongoing Covid-19 pandemic is a threat to human life around the globe, and the source of a broad spectrum of mostly negative emotions, such as fear, anger, and sadness. Furthermore, the social isolation measures recommended by the WHO and adopted by many nations impose changes on many kinds of social interactions, from more distant working relationships to closer family interactions. The planet’s population has suddenly had to adjust to a new threat and a new social reality, which puts a premium on the ability to cope with the emotional load inherent in the situation. If these challenges are processed in dreams, as proposed (with different nuances) by TST, ERT and SST, they should be more apparent in dream reports collected during the pandemic than in dream reports collected a few months before it began.

A comparison of dream reports obtained before and during the Covid-19 pandemic goes beyond trauma as separately experienced by individuals, and reflects a collective traumatic experience, as is often the case during plagues, wars and natural disasters. One of the main difficulties in trying to disambiguate the predictions made by TST, ERT and SST is that stress manifests itself in many ways, and there are paradoxical effects that must be taken into account when stress becomes excessive.

Dream reports collected from New Yorkers after the 9/11 attacks included incidents of being overwhelmed by a tidal wave or being attacked and robbed [7]. This study of an acute traumatic event with collective repercussions supports the notion that the more anxious one becomes in waking life, the more vivid the dream images will be—an interpretation consistent with ERT [2]. Potentially conflicting evidence comes from a study of dream contents produced by British officers held in a Nazi camp for prisoners of war (POW) between 1940–1942 [9] (i.e. a chronic traumatic event). POW dream reports showed more content concerning battles, imprisonment, escape, and food than the male norms from the same era. Not much social interaction of any kind was detected in these dream reports, with less friendliness, sexuality, and even less aggression than the male norms [9]. However, aggression was unusually extreme when it occurred in POW dreams, and its content was linked to previous battles rather than camp life [9]. POWs reported less good fortune or misfortune in their dreams, along with frequent bland dreams about the camp’s tedium [11]. The fact that dream reports produced by German civilians during the Nazi regime were also extremely tedious led the author to conclude that the oppressive environment was so extreme as to repress even dreaming [11]. Another study that can help understand the impact of social isolation on dreaming is a self-observational comparison of a long series of dream reports collected over several years, including long periods of social isolation from 1960 to 1963, when the dreamer was a prisoner [21]. The author concluded that interpersonal relationships diminished slowly and gradually in the impoverished prison environment, although some important social attachments remained [21].

Studies comparing dream reports from children in war zones showed a higher number of threatening events, and the severity of the reported events varied in proportion to the severity of the suffering related to the trauma [22]. Not only during the trauma, but years later, longitudinal studies indicate longlasting effects of collective trauma on dreams [23]. They reveal the persistence of nightmares for several decades following war events, even in the general population who did not serve at the front, and increasing with the dreamers’ proximity to combat [23]. These changes are accompanied by major perturbations of rapid-eye-movement sleep, which is associated with the most vivid dreaming activity [24–31].

One caveat with respect to these studies is that most data were gathered only after the person was no longer confined or had left the arena of combat [9]. Studies of dream reports collected during extended confinement, like the present study, are therefore scarce. In contrast to the British POW study [9], where dream reports were analyzed by the Hall & Van de Castle method [9], based on either positive or negative social interactions (leaving aside those without an emotional valence), in the present study we used natural language processing (NLP) tools to directly quantify (i) non-semantic structural connectedness [32–36], and (ii) emotional valences from dream reports [37–41], and to estimate (iii) semantic distances to specific probe words [42–45]. The non-semantic connectedness of word graphs is a sensitive marker of structural change in discourse [32–36], and the measurement of emotional contents has been widely applied in the detection of mental suffering [37–41]. Estimating semantic similarity based on the co-occurrence of words in a representative corpus has enabled quantitative measurement of subjective aspects of mental health expressed in memory reports [42–45]. These NLP tools offer the advantage of being objective, automated, and less affected by cultural bias.

More than just tracking signs of mental suffering, could the observation of dreams help to understand personal challenges and mitigate suffering? Blagrove and collaborators proposed that dream sharing has an empathic effect on the dreamer and on significant others engaged in a dream-telling situation [46]. Trait empathy was found to be significantly associated with the frequency of listening to the dreams of others, the frequency of telling one’s dreams to others, and one’s attitude toward dreams. Moreover, the dream-discusser’s empathy towards the dream-sharer was found to increase significantly as a result of the dream discussion, with medium effect size, whereas the dream-sharer presented a small decrease in empathy towards the dream-discusser [46]. Based on these results, the authors suggest that dreams act as a piece of fiction that can be explored by the dreamer together with other people, thus inducing empathy about the dreamer’s life circumstances [46]. They also speculate that the story-like characteristics of adult human dreams may have been positively selected during human evolution as part of the selection for emotional intelligence, empathy, and social bonding [46]. Given these results, one might argue that talking about dreams during social isolation can have a positive effect on individuals’ experiences, helping them to mitigate mental suffering, and potentially leading them to engage in other forms of creative social connection.

**Fig 1:**
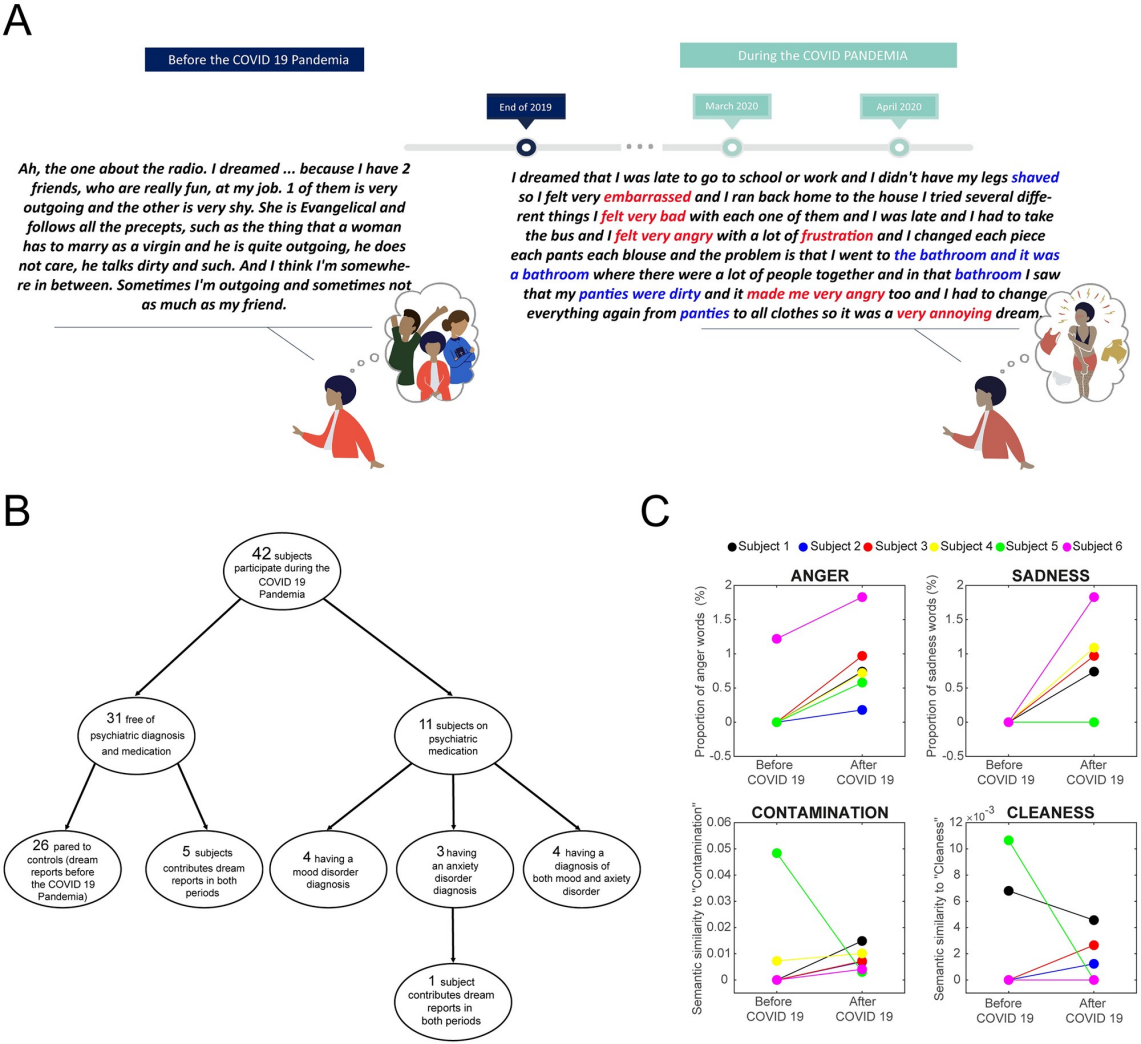
Are dream reports from the Covid-19 pandemic period different from dream reports prior to the pandemic?. (A) Data collection timeline (audio recordings of dreams reported via smartphone application), and illustrative examples of two translated dream reports by the same participant, one before and one after the Covid-19 pandemic. Words in red type are linked to emotions and words in blue type are semantically associated with the terms “contamination” and “cleanness”. (B) Flowchart showing group composition. (C) Representative examples of computational analysis of dream reports by the same participants before and after the Covid-19 pandemic.

With the aim of contributing new empirical evidence to the debate on dream function, this article presents the results obtained by using NLP tools to gauge the impact of the waking challenges associated with the Covid-19 pandemic on dreams reported by people subject to lockdown. Based on the partial overlap among TST, ERT and SST, we expected to observe the following: (1) changes in the emotional and semantic contents, and in the narrative structure, of dream reports collected from mentally healthy individuals after the Covid-19 pandemic had been declared in Brazil, in comparison with well-matched dream reports collected prior to the pandemic; (2) an association of these specific features of pandemic dreams with mental suffering, measured by psychometric scales; and (3) an association between the self-reported emotional impact of dream observation and the effect of dream telling (Fig 1).

## Results

### Comparison between pandemic and control dreams and longitudinal observation

**Fig 2:**
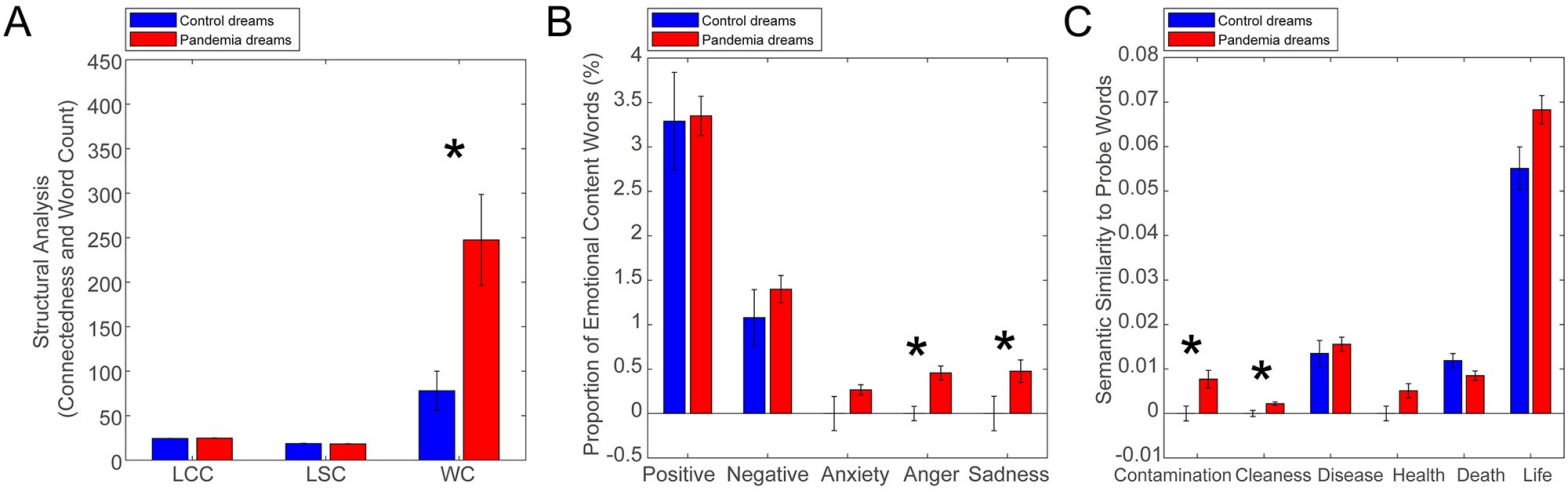
Pandemic dreams in comparison to control dreams. (A) Structural analysis is based on representation of word trajectory as a graph. No significant difference in word connectedness was found, measured by LCC or LSC. Word count was different between the groups (p < 0.0001, pandemic dreams are longer, Cohen’s d = -0.9363). * represents significant differences between groups after Bonferroni correction for multiple comparisons (p < 0.0167). Bars indicate median values and error bars indicate standard error. (B) The proportion of emotional words in dream reports. Pandemic dreams present a higher proportion of words related to Anger (p = 0.0001, Cohen’s d = -0.6937) and Sadness (p = 0.0003, Cohen’s d = -0.2156) compared to paired control dreams. * represents significant differences between groups after Bonferroni correction for multiple comparisons (p < 0.0100). Bars indicate median values and error bars indicate standard error. (C) Semantic similarity to probe words. Pandemic dreams present higher semantic similarities to the terms “contamination” (p = 0.0008, Cohen’s d = -0.4841) and “cleanness” (p = 0.0022, Cohen’s d = -0.1534). * represents significant differences between groups after Bonferroni correction for multiple comparisons (p < 0.0083). Bars indicate median values and error bars indicate standard error.

A first finding was that pandemic dream reports presented more words in general than pre-pandemic dream reports. A structural analysis of the pandemic dreams and controls, however, did not reveal any difference: although pandemic dreams presented a higher quantity of words in general, no difference was found with respect to word connectedness (Fig 2A, Table 1).

**Table 1:** Comparison of pandemic and control dream reports and correlations with report length measured by total word count.

**Wilcoxon Ranksum Comparison Pandemic x Control Dreams**						
**Semantic similarities**	**contamination**	**cleanness**	**sickness**	**health**	**death**	**life**
p (* < 0.0083)	0.0008*	0.0022*	0.1467	0.0589	0.2413	0.0738
**Emotional content %**	**positive**	**negative**	**anxiety**	**anger**	**sadness**	
p (* < 0.0100)	0.7408	0.1598	0.1069	0.0001*	0.0003*	
**Structural analysis**	**LCC**	**LSC**	**WC**			
p (* < 0.0167)	0.2975	0.0859	0.0000*			
**Spearman Correlation to WC**						
**Pandemic dream peculiarities**	**anger**	**sadness**	**contamination**	**cleanness**		
p (* < 0.0125)	0.1367	0.4164	0.3836	0.2085		
Rho	-0.27	0.15	-0.16	0.23		
**Spearman Correlation to time of dream observation (days after started the lockdown)**						
**Pandemic dream peculiarities**	**anger**	**sadness**	**WC**	**contamination**	**cleanness**	
p (* < 0.0100)	0.2323	0.6336	0.6151	0.5189	0.0291	
Rho	0.10	-0.04	0.04	0.05	0.17	

Comparisons using non-parametric Wilcoxon Ranksum test, Bonferroni corrected (p-value threshold indicated for each analysis).

* indicates results considered significant. The significant differences were used to choose the features to be correlated with word count and with time of observation (measured in days after the beginning of lockdown).

Although structure seems to be preserved, there were differences in the emotional and semantic content of dream reports in the two groups: the proportion of anger- and sadness-related words was higher for pandemic dreams. Semantic similarities to the terms “contamination” and “cleanness” were also higher (Fig 2B and 2C, Table 1). No difference was found in the proportion of positive emotions, negative emotions, or anxiety-related words, or for semantic similarities to the terms “sickness”, “health”, “death” or “life” (Fig 2B and 2C, Table 1).

The length of the pandemic dream reports could create a bias, and for that reason the structural analysis (word connectedness) was conducted in fixed windows of 30 words (see Methods). The emotional analysis was also normalized by the total number of words: the results are presented as the proportion of words, or a ratio of the total number of emotional words in the text by the text word count (see Methods). Given that semantic similarity could either increase or decrease in relation to the word count (and the relationship between word count and semantic similarity to probe words is not trivial, since only the words with a minimum similarity of 0.3 were considered), we performed a Spearman correlation analysis on this dataset, between all the measurements that revealed any difference between groups and word count (Table 1). If there were any significant associations between emotional or semantic measures and word count, this could be a potential bias explaining differences between the groups. As no correlations were found (Table 1), we conclude that the difference in dream report length was not associated with either emotional or semantic differences between pandemic and control dreams, and therefore could not account for or mitigate the findings.

We then determined whether the pandemic dream peculiarities increase or decrease over the dream observation period, by performing Spearman correlations with the dream observation period. Semantic similarities to the term “cleanness” did present an increase over time (Table 1), but Spearman correlations did not reach significance after Bonferroni correction (Rho = 0.17, p = 0.0290, Table 1).

### Association with mental suffering

**Fig 3:**
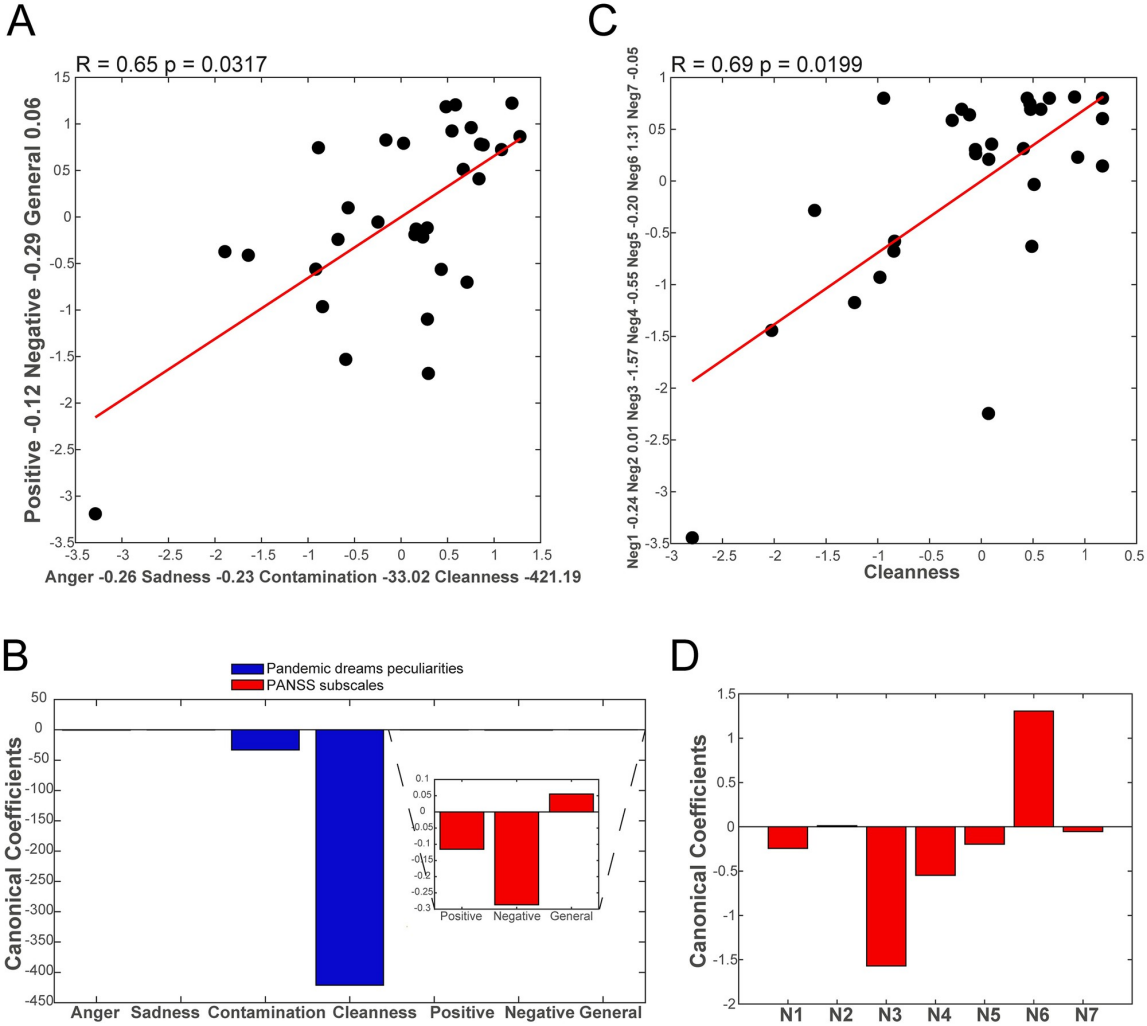
Canonical correlations of pandemic dream peculiarities and psychometric evaluation (severity of mental suffering measured by PANSS). (A) Canonical correlations of pandemic dream peculiarities (proportion of anger, sadness, and “contamination”, “cleanness” semantic similarities) and 3 PANSS subscales (Positive, Negative, and General symptoms). R and p-values in the title, and the canonical coefficients on the x and y axes. (B) Canonical coefficients of pandemic dream peculiarities (blue bars showing the higher contribution of semantic similarity to the term “cleanness” to this correlation), and of PANSS subscales (red bars showing the higher contribution of the PANSS negative subscale). (C) Canonical correlations of semantic similarity to the term “cleanness” to 7 items of the PANSS negative subscales. R and p-value on the title, and the canonical coefficient on x and y-axis. (D). Canonical coefficients of the PANSS negative subscale (red bars showing the higher contribution of PANSS negative subscale N3 items—Poor Rapport, N6 items—Lack of Spontaneity, N4 items—Passive Social Withdrawal). The canonical coefficient of semantic similarity to the term “cleanness” is -483.50.

Pandemic dream peculiarities such as the proportion of anger- or sadness-related words, and semantic similarities to “contamination” and “cleanness” were combined and correlated, using canonical correlation, scored according to PANSS (a psychometric scale that evaluates positive symptoms linked to psychosis such as hallucinations and delusions; negative symptoms linked to social isolation and cognitive impairment; and a general subscale linked to non-specific mental suffering such as mood and anxiety symptoms) (Fig 3). We found a significant correlation between the combination of dream content peculiarities and mental suffering (Fig 3A). PANSS negative subscale and semantic similarity to the term "cleanness" contributed higher coefficients to the canonical correlation, and both co-varied in the same direction (the higher the semantic similarities to “cleanness” in the dream report, the higher the severity of negative subscales in PANSS (Fig 3B). This result was confirmed by multilinear correlations between the combination of pandemic dream peculiarities and the PANSS negative subscale or mental suffering related to social isolation (R^2^ = 0.3955, p = 0.0088, Coefficients: 1.3 Anger proportion + (-0.043) Sadness proportion + 18 “Contamination” similarity + 984.79 “Cleanness” similarity + 7.7). In other words, pandemic dream peculiarities accounted for 40% of the PANSS negative subscale variation. To better understand which items were particularly related to “cleanness”, we calculated the canonical correlations between all seven items of the PANSS negative subscale and semantic similarity to the term “cleanness” (Fig 3C). We found that most of the variance of this correlation was due to co-variance between the semantic similarity of dream reports to the term “cleanness” and the N3 items of the PANSS negative subscale (poor rapport), with a smaller contribution in the same direction with the N4 item (passive social withdrawal), and in the opposite direction with the N6 item (lack of spontaneity in talking). In other words, the more similar to cleanness the dream report was, the greater the severity of poor rapport and passive social withdrawal, and the greater the participant’s spontaneity in talking during the interview.

We performed the same multilinear correlational analysis for a group of participants under psychotropic medication that self-reported mood and/or anxiety disorder. No significant correlation was found between peculiarities of pandemic dreams and psychometric evaluation, perhaps because the sample size was too small to reach a significant level (Total PANSS: R^2^ = 0.30, p = 0.8458, Positive Subscale: R^2^ = 0.68, p = 0.3675, Negative Subscale: R^2^ = 0.26, p = 0.8843, General Subscale: R^2^ = 0.19, p = 0.9369). Interestingly, however, the association with the positive subscale was the strongest found: a pattern that differs from that of the group without diagnosed mental disorders.

### Association with the affective impact of dream observation and dream telling (self-evaluation scale)

**Fig 4:**
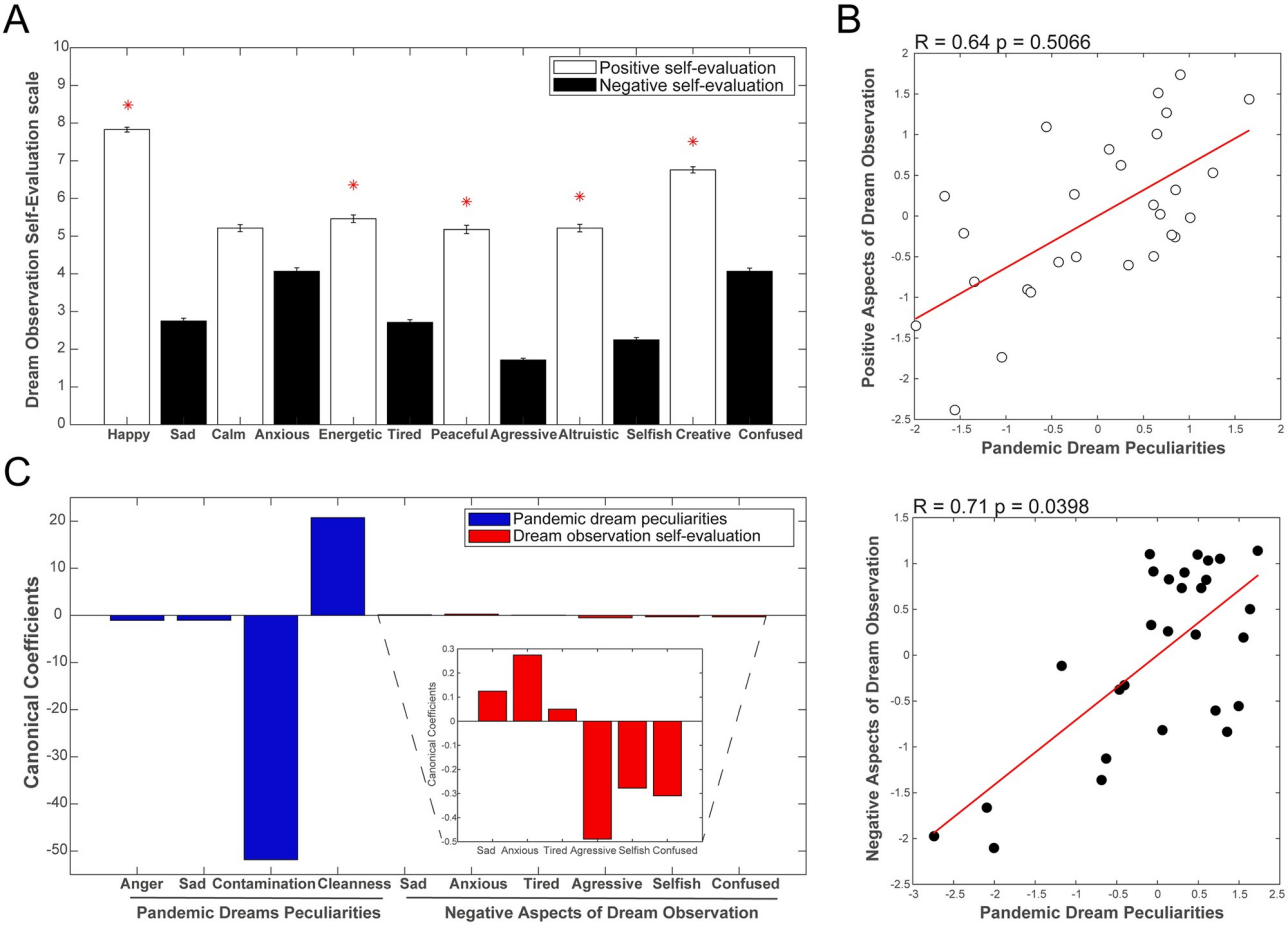
Self-evaluation of dream observation/telling. (A) Comparison between positive and negative aspects of self-evaluated dream experience. We took into consideration how participants related positive aspects (e.g. happy, calm, energetic, peaceful, altruistic and creative) to their dream-observation/telling experience during the first month of lockdown due to the Covid-19 pandemic, in contrast to negative aspects (e.g. sad, anxious, tired, aggressive, selfish and confused). (B) Canonical correlations between pandemic dream peculiarities (Proportion of Anger- and Sadness-related words + Semantic similarities to the terms “contamination” and “cleanness”) and positive (top) or negative (bottom) self-evaluated aspects of dream observation/telling. R and p values are presented in the title. (C) Canonical coefficients of pandemic dream peculiarities (blue bars, showing the higher contribution of semantic similarity to the term “contamination” to this correlation), and negative aspects of dream observation (red bars).

To identify a potential affective impact of the dream observation and dream telling process, at the end of the data collection period, we asked participants to complete a self-evaluation questionnaire in which they rated, using a 1 to 10 scale, the degree to which the dream observation/telling experience increased their states of feeling happy/sad, calm/anxious, energetic/tired, peaceful/aggressive, altruistic/selfish, and creative/confused (the questionnaire established pairs of positive/negative subjective evaluations). First, we compared the scores assigned by participants to determine how they self-evaluated the dream-observation/telling experience in positive and negative terms. We found higher positive evaluation for almost all the pairs of subjective impact analyzed (more happy than sad, more energetic than tired, more peaceful than aggressive, more altruistic than selfish, and more creative than confused) (Fig 4A). There was no difference between calm and anxious (Fig 4A). We then correlated all pandemic dream peculiarities to the positive and negative self-evaluated aspects and found a significant canonical correlation to negative aspects of dream observation/telling (Fig 4B). In addition, while analyzing canonical coefficients, we noticed that most of the correlation variance is explained by the co-variance of semantic similarity to the term “contamination” with scores on self-evaluation for aggressiveness (i.e. the more similar to “contamination” the semantic content of the dream report, the higher the feeling of being aggressive, selfish and/or self-confused) (Fig 4C). In contrast, the higher the similarity to “cleanness”, the lower the scores for aggressiveness, selfishness or self-confusion, and a higher score for anxiety) (Fig 4C).

## Discussion

Overall, our results are consistent with the hypothesis that dreams during the lockdown period reflect the waking challenges presented by the Covid-19 pandemic. Consistently with the Emotional Regulation Theory [2], negative emotions such as anger and sadness are more prominent during the pandemic period, reflecting a higher emotional load to be processed. Furthermore, in accordance with the Threat Simulation Theory [3], we found a higher semantic similarity to “contamination” and “cleanness”, reflecting the fear of contamination and the processing of new strategies to avoid infection by the virus. Moreover, there are points of convergence between our findings and the Social Simulation Theory [4], in the sense that the significant similarity to “cleanness” in dream reports points towards new social strategies (e.g. use of masks, avoidance of physical contact) and new hygiene practices (e.g. use of hand sanitizer and other cleaning products) that have become central to new social rules and behavior. Taken together, these findings seem to show that dream contents reflect the different sources of fear and frustration arising out of the current scenario, which involve health-related and economic fears, social distancing from relatives, friends, and peers, and the simulation of new strategies to overcome health-threatening events and to adjust to new social rules.

Interestingly, our account of pandemic dream reports reflects changes in habits more than concerns over pandemic-related events that would presumably have a greater negative impact, such as the death of relatives or friends. There is a consistent similarity between dream contents and daily concerns, with concrete changes in habits related to contamination and cleanness, which tends to increase over the course of the lockdown. These are changes that are discussed and adopted worldwide [47]. Nevertheless, during the first month of observation, disease and fear of death did not represent an actual challenge for most of the participants (during the follow-up, only one participant confirmed a Covid-19 infection, and another reported a close relative with a confirmed infection). Instead, the daily routine of individuals in our study during this initial period was concretely affected by changes of habits and fear of contamination. The results may be quite different at later time points, when sickness and death are more likely to make themselves felt in the participants’ personal lives. A follow-up study with the same participants is now underway.

Although the participants in our sample did not experience any severe individual trauma by reason of fear of death or grief at losing a loved one or a job, the change was, in reality, already enough for some individuals to experience mental suffering. Adjustment to new social rules rather than the threat of the virus *per se* was the major driving force behind mental suffering and the changes in dream contents in our study. The combination of dream content changes (higher proportion of anger and sadness combined with higher semantic similarity to “contamination” and “cleanness”) was significantly correlated with mental suffering related to aspects of social isolation, reflecting 39% of PANSS negative subscale variance. The mental suffering associated with poor rapport and passive social withdraw, combined with more fluency while talking during the interview, was associated with higher similarity in dream content to the term “cleanness”. Such suffering was not severe to the point where it could be considered pathological, but the new reality imposed by the pandemic forced people to attend to many urgent new demands. Some individuals were less resilient than others, and this variation was detectable in dream reports. Interestingly, the individuals whose dreams reflected more evidence of adaptative mechanisms were also those presenting the highest levels of mental suffering, but a subsample of individuals with a diagnosis of mood and/or anxiety disorder did not show the same level of significant association with that class of symptoms. Although the sample size of this group is too small for the results to be conclusive, the sizable correlation coefficients suggest that there might be a real association, emphasizing the importance of increasing the sample size in further studies. Given that psychotropic medications have been reported to exert a major impact on dreaming [48, 49], one can speculate that they could be conflating the results of this subsample in our study.

During the pandemic period the dream reports were longer and wordier than before the pandemic. This may indicate that people are paying more attention to dream experiences at this moment in time, but it might just as well suggest an increase in the ability to remember dream details because of the possibility that participants were staying longer in bed in the morning during lockdown, although this factor did not interact with other results (i.e. correlations with word count were not significant). Importantly, changes in non-semantic structural connectedness were not observed. Such changes have been previously associated with cognitive impairments in psychosis [32, 34, 35] or during typical cognitive development [33, 50, 51], and therefore were not expected to be found in healthy adult individuals such as those sampled here.

Notably, the process of observing and reporting dreams was positively evaluated by participants. This may indicate a relatively safe way for self-observation and mental health management that can be recommended during this period of uncertainty in waking life [46]. The simple act of observing and reporting dreams can have a positive impact on mental health [46]. The peculiarities of the dream reports sampled in this study signal the need to be cautious: the more similar the dream reports were to the term “contamination”, the more people reported feeling aggressive, selfish, or confused after dream observation; in contrast, the more similar the dreams were to the term “cleanness”, the higher the feeling of anxiety after dream observation. Dream observation thus seems to be quite informative about mental health issues.

The present study of the impact of the Covid-19 pandemic on dreaming is preliminary, and several limitations should be mentioned. The first is the small sample size and the lack of representative diversity in gender balance, age, and educational level. Another limitation was the recruitment process, which differed for the two groups, due to the restrictions imposed by the pandemic. The control group was recruited more randomly than the pandemic group, since during the pandemic the participants who were more interested in the topic of this survey were more willing to accept the invitation to participate. Furthermore, given that the original study design did not include pandemic dreams, we did not collect dream recall rates in the first period of data collection. Another important limitation is that semantic similarities were not directly controlled for word count, as were all the other measures. There was no correlation between word count and semantic similarities (for the terms “contamination” and “cleanness)”. This means that the increased similarities to those contents during the pandemic period are not simply explained by verbosity. Notwithstanding, the relationship between semantic measures and verbosity is not trivial, and future studies shall take this into consideration. In addition, there was no separate measurement of social isolation, since the measurement we employed in the study reflected mental suffering related to social interactions, using the PANSS scales. Thus, the conclusions on this aspect should be viewed with caution, and social isolation should be better controlled in future studies.

Although these limitations might prevent the generalization of the results of this study to other populations, our work does suggest a relatively simple and safe manner to track mental suffering during periods of imposed social isolation. Larger studies with diverse samples should improve our understanding of dream reports as privileged windows into our inner suffering and fears. They might also help us to identify early signs of mental suffering, playing a key role in the prevention of the most severe consequences for mental health. Observing and telling dreams may help individuals to overcome challenges [46], flagging feelings such as anxiety and sadness. The present study on dream reports during the Covid-19 pandemic supports the general notion that dreams reflect daily changes during critical periods of our lives, in association with mental suffering.

## Methods

### Participants

To address the three hypotheses formulated, we followed 42 participants during the initial days of lockdown in Brazil, after the announcement of the Covid-19 pandemic by the World Health Organization WHO (from March 12 to April 21, 2020) (Fig 1B and Table 2). The participants were invited to join the study either because they had already participated in other data collection phases (dreams collected before the Covid-19 pandemic), or because they were a good match to become a member of the control group (participants who were already in a volunteer database maintained by our laboratory that matched the control subjects for age, educational level and sex).

**Table 2:** The pandemic and control groups were balanced for years of age, formal education, and sex.

	Age (years)	Education (years)	Sex
**Pandemic dreams group**	34 ± 8.72	16 ± 2.39	5M/26F
**Control dreams group**	28.5 ± 9.13	16 ± 1.50	6M/25F
**Pandemic x Control (p value)**	0.0665	0.4019	0.7396

Age and education show median, standard deviation, and p-value for Wilcoxon Ranksum comparisons. Sex shows the number of males (M) and females (F) and p-value after Chi-square comparison between groups.

Considering the entire period of observation (from March 12th to April 21st 2020), this group had on average 4.88 dream reports ± 4.37 (mean ± standard deviation), with a minimum of 1 dream report and a maximum of 18 dream reports collected during the 47 days of observation). Considering only the nights when the participants recalled at least one dream, the group presented on average a rate of 1.33 dream recalls per night, ± 0.50 (mean ± standard deviation), with a minimum of 1 dream recall per night and a maximum of 3.25 dream recalls per night.

As 11 of them reported continuous use of psychotropic medication and a mental health diagnosis, we analyzed their data separately (Fig 1B). The main sample was composed of a control group of 31 participants paired to the 31 participants without a mental health diagnosis (Fig 1B and Table 2). All the control participants were also free of mental health symptoms, diagnosis, and psychotropic medication. Participants in both groups used the same method of dream collection reports before and during the Covid-19 pandemic (a customized smartphone application designed to record oral dream memory reports for subsequent tracking of signs of mental suffering). Six of the 31 participants in the control group were also able to participate in the pandemic period of data collection (Fig 1B), and these six paired examples of pre- and post- pandemic dream reports are shown in Fig 1C. Median and standard deviation in age and educational level are summarized in Table 2, along with sex distribution. All the participants agreed to participate and signed a document giving their informed consent. The study was approved by the Federal University of Rio Grande do Norte Research Ethics Committee (permit # 3.613.186).

### Protocol

Prior to the pandemic period, during September and November 2019, the participants were invited to test a smartphone application designed to collect remotely oral memory reports that would subsequently be used to track mental suffering. The application prompted participants to report a dream that had to be at least 30 seconds long. This was the first question presented and the volunteers were instructed to use the application as soon as they woke up. As a subsequent step, the application requested a report from the previous day, and presented three images, based on which the participants were asked to tell a story. All the audio files were immediately and securely transferred to the university server, and transcribed into text files by members of the study team. In this phase, we focused on the pre-pandemic dream memory reports, which were later compared to pandemic dream reports. A total of 31 pre-pandemic dream reports were generated.

During the pandemic period, prior to reporting their dreams, the participants had first to answer an online survey related to general information, regarding sex, age, educational level, family income, sleep patterns, and mental health diagnosis and/or medication use. They were instructed to record their dreams daily in an audio file and share it with the researchers, who would transcribe it to text files. A total of 208 dream reports from this period (the first month of the Covid-19 pandemic) were generated and averaged per subject for comparison with paired controls.

At the end of the pandemic observation period, a trained psychiatrist applied the PANSS scale to measure the severity of mental suffering related to the pandemic period. The PANSS scale is divided into three subscales and the major focus is on psychosis. The first subscale is composed of seven questions and is referred to as the positive symptoms scale, which focuses on symptoms such as delusions and hallucinations; the second is also composed of seven questions and is refered to as the negative subscale, which is related to symptoms associated with social withdraw, isolation, cognitive impairment, and recursive thinking; the last subscale is composed of 16 general questions related to mood and anxiety symptoms such as tension, depression, panic attacks, time/space orientation, or lack of judgment. All questions are graded from 1 to 7.

The final procedure was to have participants complete an online survey, in which they self-evaluated the subjective impact of dream observation and dream telling during th pandemic period. The scale requested the participants to grade from 1 to 10 how the dream observation experience had made them feel: happy, sad, anxious, calm, energetic, tired, peaceful, aggressive, altruistic, selfish, creative, or confused.

### Text analysis

After transcribing all the words in the dream reports, we performed three different computational language analyses, using different strategies:

#### Structural analysis using SpeechGraphs software [35]

For this analysis, each word in the text file was considered as a node and the temporal sequence between words was represented as directed edges. Here we focused on the total word count (WC) and connectedness attributes (LCC—the number of nodes in the largest connected component and LSC—the number of nodes in the largest strongly connected component), as these attributes were the ones most associated with mental health disorder (such as schizophrenia [32, 34–36]). As there was no maximum limit for oral dream reports, to control for word count differences, we analyzed graphs of 30 words, using a step of one word to plot the next graph [35]. We used a sliding window technique, in which we chose an initial set of 30 words, plotted a graph, moved to the next word and plotted the next graph with the following set of 30 words, and so on consecutively, until the complete set of 30 words in the text was graphed. This allowed us to screen the entire text in 30-word consecutive graphs. We then calculated the LCC and LSC of all 30 word-graphs and averaged all LCCs and LSCs from the same reports.

#### The proportion of words using LIWC with the Brazilian Portuguese Dictionary

We used the LIWC software [52], which searched and classified words in the dream reports according to a validated Brazilian Portuguese Dictionary [53] to find words with emotional content (positive emotions, negative emotions, anxiety, anger, and sadness related words). The software returns the proportion of words with the specified content in the dream reports. As it is already a proportion, the result is controlled by word count differences.

This dictionary is composed of a set of words considered to be related to some category. The words “love”, “nice” and “sweet”, for example, are linked to positive emotions. A total of 406 words are included in the positive emotional set, and 499 in the negative emotional set (subdivided into three sets: Anxiety, with 91 words such as “worried” and “nervous”; Anger, with 184 such as “hate”, “kill” and “annoyed”; and Sadness with 101 words such as “crying”, “grief” and “sad”) [37, 40]. The process of building the dictionary relied on inter-rater validity and reliability, evaluated and replicated [37–39], from the time it was first proposed in 1992 and 1994 to the 1997 and 2007 versions [37, 40]. Numerous publications have used this method to evaluate emotional tone, as detailed in [40].

In this study, we calculated inter-rater reliability by having 20% of the sample of dream reports classified as having Anger or Sadness according to the judgment of two independent human raters who were blind to group and LIWC evaluation. The human raters had to grade from 1 to 5 the emotional content of the text (the amount of “Anger” and “Sadness”). If they graded 1, we considered that the text did not contain emotional content; if the text was graded from 2 to 5, we considered that the text did contain emotional content. We then used a Naïve Bayes classifier to determine whether it was possible to classify the text as containing, or not, emotional content based on the LIWC evaluation, based on the human raters’ assessment. In other words, we used the LIWC evaluation (proportion of emotional content in the text) as input for the machine learning classifier, and labels were the human raters’ evaluation (based on the presence or absence of emotional content). For Anger evaluation, the Naïve Bayes classifier produced the following results: a mean accuracy in classification of 86.46% correct and 13.54% incorrect (Rater 1: 91.67% correct and 8.33% incorrect; Rater 2: 81.25% correct and 18.75% incorrect). For Sadness evaluation, the results were a mean accuracy of 81.25% correct and 18.75% incorrect (Rater 1: 89.58% correct and 10.42% incorrect; Rater 2: 72.92% correct and 27.08% incorrect).

#### Semantic similarity using customized software to perform the Fast text method in Python

To investigate how dream content reflected pandemic-related concerns and changes in daily habits, we estimated the semantic similarity of dream reports to the probe words (“contamination”, “cleanness”, “sickness”, “health”, “death”, “life”—originals in Portuguese: “contaminação”, “limpeza”, “doença”, “saúde”, “morte”, “vida”) using word embedding techniques. Word embedding is based on a distributional hypothesis, which states that semantically similar words tend to appear in similar contexts [54]. Given that the reports were in Portuguese, we used the Fast text pre-trained multilingual word embedding model [55, 56]. This model is built using a sliding window of predefined length that moves along a Portuguese Wikipedia corpus. At each step, a neural network is trained to predict the central word from the context, i.e. from the other words in the window. Once the neural network is trained, a vector representation for each word in the corpus is extracted from the input and output layers of the model, which is used as the word embedding. The original text files were transformed to lowercase, word-tokenized, and cleared of non-alphabetic tokens and stop-words (using nltk Portuguese stop-word list [57]). Then, for each text file, we calculated the semantic similarity of each word to the probe words by calculating the cosine between the word and the probe word. To control for the possible difference in word count, and to refine the results to reflect only the words most related to the probe terms, for the final average similarity analysis we considered only those words that reached a threshold of 0.3 (the maximum similarity is 1). All the words that reached this threshold were included and averaged to compose a final semantic similarity analysis of the dream report to the probe words.

$$Semantic\;Similarity\;of\;a\;dream\;to\;a\;probe\;word=average\;(cos(v_{1\;to\;N},v_{2}))$$

where |**v**_**2**_| refers to the probe words (“contamination”, “cleanness”, “sickness”, “health”, “death”, “life”) and |**v**_**1** to N_| refers to the all the words in dream reports that presented a similarity of at least 0.3 to the probe word in question.

In light of the effects that the Covid-19 pandemic might have on different degrees of mental suffering, and expecting first an initial stage of fear, we predicted that the dreams would present a higher level of negative emotional words (related to anger, sadness, and anxiety), associated with a semantic content related to the fear of contamination (or, in contrast, increase of cleanness content, reflecting a strategy to avoid contamination and adjust to this novel social demand). At a further level of mental and physical suffering (mainly for those who were infected or knew someone who had been infected), we predicted an increase of semantic content related to sickness (or, in contrast, content also related to health, in search of healing). To conclude, for those who had lost a family member or friend, we predicted semantic content related to death (and also to life, as a contrasting way to engage survival mechanisms). In this specific case, a higher level of mental suffering could impact the mental organization and verbal memory process, affecting dream reports connectedness (LCC and LSC). However, we were not expecting this to occur, as the subjects had not been diagnosed with previous mental disorders (such as psychosis).

### Statistical analysis

To address the first hypothesis, we compared the dream reports produced during the Covid-19 pandemic period (Pandemic Dreams) to dream reports produced before the outbreak of Covid-19. To that end, we averaged all dream reports from the same participant produced during the pandemic period, so as to have only one data point for each participant.

As the data was not normally distributed (all the text analyses were tested for normality distribution using the Kolmogorov Smirnoff test and all p values were lower than 0.0001), we used a nonparametric test, Wilcoxon Ranksum. We stratified the analysis as structural analysis (three attributes: WC, LCC, and LSC), analysis of the proportion of emotional content (five attributes: positive emotions, negative emotions, anxiety, anger, and sadness related words) and semantic similarity analysis to six probe words (“contamination”, “cleanness”, “sickness”, “health”, “death”, “life”). For each analysis, we corrected for multiple comparisons, using the Bonferroni test, which reset significance of p-value to lower than 0.0167 for structural analysis, 0.0100 for analysis of the proportion of emotional content, and lower than 0.0083 for semantic similarity to probe words.

For the longitudinal analysis, we correlated only the dream peculiarities found in the previous comparison, and correlated to the time period of dream observation counted in days. As we found five attributes, we corrected for multiple comparisons using the Bonferroni method and considered difference p values lower than 0.0100.

To investigate the association between dream content and mental suffering, we combined and correlated all the pandemic dream content differences found with the PANSS subscales (positive, negative and general) using canonical correlations, and confirmed the results by performing a multilinear correlation between the PANSS subscale with the highest canonical coefficient and the pandemic dreams variables. To better understand which symptoms were leading the correlations, we performed a canonical correlation between all questions in the PANSS subscale and the pandemic dreams variable that showed the higher canonical coefficient. To investigate the group of individuals having a psychiatric diagnosis and under medication, we performed only the multilinear correlation (to avoid overfitting combining multiple variables, as this group was too small).

To examine the relationship between the self-evaluation of dream observation and dream telling and dream report peculiarities, we first compared six positive aspects of the self-evaluation survey to the six paired negative aspects of the survey, correcting the results for multiple comparisons using the Bonferroni method (considering significant p < 0.0083). We then correlated the positive and negative aspects with identified pandemic dream peculiarities using canonical correlation, and studied the coefficients from the two sets of variables, to identify those that co-varied.

We used canonical correlations (which is a general procedure for investigating the correlation between two sets of variables). The reasoning here was to discover whether there could be shared information between dream peculiarities that could help explain the variation in the final psychometric evaluation or the self-evaluated experience of dream observation and telling. We also analyzed the canonical coefficients to gain a deeper understanding of each variable contribution to that association.

## Supporting information

S1 Table Computational language analysis applied to dream reports. Raw data from participants that reported a dream before the Covid-19 outbreak (identified as controls—Ctr) and after the announcement of the Covid-19 pandemic by the WHO (identified as subjects—Sub). Emotional analysis computed as the proportion of words with specified content; structural analysis computed as the number of nodes in the LCC or LSC (connected components of speech graphs) or the word count, and semantic analysis computed as average cosine between the words in dream reports and the probe words identified at the top of the table. For dream reports from the pandemic period, all the measures were average per subject if the same participant reported more than one dream in the follow-up. For more details, read Methods.
(DOCX)

S2 Table Psychometric evaluations of participants during the pandemic period of observation. PANSS psychometric scale is composed of 30 questions, graded 1 to 7 for each question, and subdivided into 3 sub-scales: positive symptoms, negative symptoms, and general symptoms. More details in Methods.
(DOCX)

S3 Table Self-evaluation of the dream observation experience during the first month of social isolation. The participants were asked to grade from 1 to 10 how they felt after the dream observation experience. Details in Methods.
(DOCX)
