# Supplementary material for: Dreaming during the Covid-19 pandemic: Computational assessment of dream reports reveals mental suffering related to fear of contagion
Source: PLoS One. 2020 Nov 30;15(11):e0242903. doi: 10.1371/journal.pone.0242903 (PMC7703999; doi:10.1371/journal.pone.0242903)
Supplement: S1 Table — Raw data from participants that reported a dream before the Covid-19 outbreak (identified as controls—Ctr) and after the announcement of the Covid-19 pandemic by the WHO (identified as subjects—Sub). Emotional analysis computed as the proportion of words with specified content; structural analysis computed as the number of nodes in the LCC or LSC (connected components of speech graphs) or the word count, and semantic analysis computed as average cosine between the words in dream reports and the probe words identified at the top of the table. For dream reports from the pandemic period, all the measures were average per subject if the same participant reported more than one dream in the follow-up. For more details, read Methods. (DOCX) [file pone.0242903.s001.docx]

**S1 Table:** Computational language analysis applied to dream reports. Raw data from participants that reported a dream before the Covid-19 outbreak (identified as controls – Ctr) and after the announcement of the Covid-19 pandemic by the WHO (identified as subjects – Sub). Emotional analysis computed as the proportion of words with specified content; structural analysis computed as the number of nodes in the LCC or LSC (connected components of speech graphs) or the word count, and semantic analysis computed as average cosine between the words in dream reports and the probe words identified at the top of the table. For dream reports from the pandemic period, all the measures were average per subject if the same participant reported more than one dream in the follow-up. For more details, read Methods.

| **ID** | **Emotional Analysis** | | | | | **Structural Analysis** | | | **Semantic Similarity Analysis** | | | | | |
| --- | --- | --- | --- | --- | --- | --- | --- | --- | --- | --- | --- | --- | --- | --- |
|  | **Positive** | **Negative** | **Anxiety** | **Anger** | **Sadness** | **LCC** | **LSC** | **WC** | **Contamination** | **Cleanness** | **Disease** | **Health** | **Death** | **Life** |
| Sub001 | 3.60 | 1.77 | 0.48 | 0.70 | 0.54 | 25.47 | 18.18 | 120.33 | 0.01 | 0.00 | 0.02 | 0.00 | 0.01 | 0.06 |
| Sub002 | 3.31 | 0.92 | 0.18 | 0.18 | 0.00 | 24.01 | 18.63 | 506.00 | 0.01 | 0.00 | 0.01 | 0.00 | 0.00 | 0.08 |
| Sub003 | 3.38 | 2.08 | 0.15 | 0.72 | 0.95 | 24.68 | 18.50 | 685.50 | 0.01 | 0.00 | 0.02 | 0.00 | 0.01 | 0.08 |
| Sub004 | 3.83 | 1.29 | 0.41 | 0.32 | 0.38 | 24.30 | 19.03 | 177.92 | 0.00 | 0.00 | 0.02 | 0.00 | 0.01 | 0.08 |
| Sub005 | 2.25 | 0.58 | 0.00 | 0.29 | 0.00 | 24.82 | 19.41 | 235.50 | 0.00 | 0.00 | 0.02 | 0.01 | 0.01 | 0.12 |
| Sub006 | 4.60 | 1.58 | 0.19 | 1.01 | 0.86 | 24.35 | 18.81 | 641.67 | 0.00 | 0.00 | 0.02 | 0.01 | 0.01 | 0.07 |
| Sub007 | 3.60 | 3.09 | 1.20 | 0.55 | 1.80 | 24.87 | 17.34 | 165.67 | 0.03 | 0.01 | 0.02 | 0.01 | 0.01 | 0.07 |
| Sub008 | 2.79 | 0.88 | 0.21 | 0.00 | 0.67 | 24.51 | 19.03 | 149.40 | 0.00 | 0.00 | 0.01 | 0.00 | 0.01 | 0.06 |
| Sub009 | 4.18 | 2.28 | 0.40 | 0.58 | 0.84 | 24.99 | 18.42 | 162.40 | 0.01 | 0.00 | 0.02 | 0.00 | 0.01 | 0.09 |
| Sub010 | 3.95 | 2.63 | 1.32 | 1.32 | 0.00 | 27.83 | 17.17 | 31.00 | 0.01 | 0.00 | 0.02 | 0.01 | 0.02 | 0.03 |
| Sub011 | 6.52 | 0.54 | 0.00 | 0.29 | 0.00 | 25.57 | 19.22 | 117.00 | 0.00 | 0.00 | 0.02 | 0.01 | 0.01 | 0.06 |
| Sub012 | 3.61 | 3.92 | 0.00 | 0.31 | 3.31 | 22.53 | 18.11 | 247.50 | 0.00 | 0.00 | 0.01 | 0.01 | 0.00 | 0.06 |
| Sub013 | 2.44 | 2.44 | 1.22 | 1.22 | 0.00 | 24.02 | 12.33 | 74.00 | 0.06 | 0.00 | 0.02 | 0.01 | 0.03 | 0.04 |
| Sub014 | 2.95 | 1.47 | 0.00 | 1.05 | 0.63 | 24.88 | 18.32 | 457.00 | 0.01 | 0.00 | 0.02 | 0.02 | 0.00 | 0.05 |
| Sub015 | 4.54 | 1.08 | 0.28 | 0.46 | 0.16 | 24.38 | 19.34 | 206.50 | 0.01 | 0.00 | 0.01 | 0.01 | 0.01 | 0.08 |
| Sub016 | 2.25 | 1.40 | 0.00 | 0.84 | 0.56 | 25.41 | 18.78 | 339.00 | 0.01 | 0.00 | 0.01 | 0.00 | 0.01 | 0.07 |
| Sub017 | 3.22 | 0.96 | 0.21 | 0.22 | 0.42 | 23.65 | 18.46 | 330.53 | 0.00 | 0.00 | 0.01 | 0.01 | 0.01 | 0.05 |
| Sub018 | 2.99 | 0.75 | 0.00 | 0.75 | 0.00 | 24.84 | 17.65 | 124.00 | 0.01 | 0.00 | 0.06 | 0.05 | 0.01 | 0.04 |
| Sub019 | 3.18 | 1.19 | 0.32 | 0.57 | 0.38 | 24.61 | 18.50 | 389.83 | 0.00 | 0.00 | 0.02 | 0.01 | 0.01 | 0.09 |
| Sub020 | 5.50 | 1.73 | 0.00 | 0.86 | 0.86 | 24.98 | 19.03 | 76.00 | 0.01 | 0.00 | 0.02 | 0.00 | 0.01 | 0.09 |
| Sub021 | 3.35 | 0.68 | 0.11 | 0.11 | 0.06 | 24.36 | 17.18 | 1656.00 | 0.01 | 0.00 | 0.01 | 0.01 | 0.01 | 0.08 |
| Sub022 | 2.67 | 2.19 | 1.10 | 0.16 | 0.63 | 25.58 | 19.08 | 228.50 | 0.01 | 0.00 | 0.01 | 0.01 | 0.01 | 0.05 |
| Sub023 | 2.76 | 2.33 | 0.28 | 0.47 | 0.36 | 24.98 | 18.35 | 216.50 | 0.02 | 0.01 | 0.01 | 0.01 | 0.00 | 0.06 |
| Sub024 | 4.12 | 1.19 | 0.28 | 0.30 | 0.37 | 24.88 | 17.50 | 329.00 | 0.01 | 0.00 | 0.02 | 0.00 | 0.02 | 0.08 |
| Sub025 | 2.16 | 3.04 | 0.87 | 1.28 | 1.54 | 24.05 | 18.82 | 216.00 | 0.00 | 0.00 | 0.03 | 0.01 | 0.02 | 0.08 |
| Sub026 | 2.98 | 1.34 | 0.30 | 0.14 | 0.64 | 24.78 | 18.25 | 294.80 | 0.01 | 0.00 | 0.02 | 0.01 | 0.01 | 0.07 |
| Sub027 | 2.89 | 3.69 | 0.41 | 1.80 | 0.59 | 24.65 | 17.35 | 242.00 | 0.00 | 0.00 | 0.01 | 0.00 | 0.00 | 0.08 |
| Sub028 | 4.74 | 2.82 | 0.14 | 0.37 | 2.19 | 24.98 | 18.17 | 454.64 | 0.01 | 0.00 | 0.01 | 0.00 | 0.01 | 0.06 |
| Sub029 | 5.80 | 4.69 | 0.35 | 2.29 | 1.40 | 25.14 | 12.25 | 146.50 | 0.01 | 0.00 | 0.01 | 0.01 | 0.02 | 0.08 |
| Sub030 | 3.66 | 1.40 | 0.46 | 0.42 | 0.49 | 23.35 | 18.02 | 471.23 | 0.01 | 0.00 | 0.02 | 0.00 | 0.01 | 0.09 |
| Sub031 | 2.87 | 0.99 | 0.14 | 0.09 | 0.48 | 24.10 | 18.92 | 336.43 | 0.01 | 0.00 | 0.01 | 0.00 | 0.00 | 0.06 |
| Sub032 | 3.18 | 1.20 | 0.40 | 0.33 | 0.48 | 23.87 | 17.07 | 274.83 | 0.01 | 0.00 | 0.03 | 0.00 | 0.01 | 0.07 |
| Sub033 | 2.71 | 1.44 | 0.27 | 0.18 | 0.73 | 25.10 | 17.69 | 316.50 | 0.02 | 0.00 | 0.01 | 0.00 | 0.01 | 0.07 |
| Sub034 | 5.61 | 1.71 | 0.68 | 0.62 | 0.33 | 25.24 | 16.39 | 180.78 | 0.01 | 0.00 | 0.02 | 0.01 | 0.01 | 0.07 |
| Sub035 | 6.67 | 2.16 | 0.68 | 0.20 | 1.03 | 23.23 | 18.68 | 131.00 | 0.00 | 0.00 | 0.02 | 0.01 | 0.02 | 0.09 |
| Sub036 | 3.96 | 1.30 | 0.00 | 0.56 | 0.74 | 25.14 | 18.80 | 328.00 | 0.00 | 0.00 | 0.01 | 0.00 | 0.00 | 0.06 |
| Sub037 | 3.59 | 2.39 | 0.80 | 0.00 | 0.40 | 24.80 | 17.39 | 235.00 | 0.02 | 0.00 | 0.04 | 0.01 | 0.00 | 0.07 |
| Sub038 | 0.78 | 2.20 | 0.00 | 1.64 | 0.00 | 23.56 | 15.80 | 125.50 | 0.01 | 0.01 | 0.01 | 0.00 | 0.00 | 0.13 |
| Sub039 | 4.32 | 1.02 | 0.14 | 0.30 | 0.22 | 24.05 | 17.84 | 420.88 | 0.00 | 0.00 | 0.02 | 0.01 | 0.01 | 0.08 |
| Sub040 | 3.15 | 0.93 | 0.58 | 0.00 | 0.00 | 27.25 | 16.64 | 167.50 | 0.01 | 0.00 | 0.01 | 0.01 | 0.01 | 0.05 |
| Sub041 | 3.39 | 3.72 | 0.64 | 1.91 | 1.20 | 24.23 | 17.82 | 285.50 | 0.01 | 0.01 | 0.02 | 0.01 | 0.01 | 0.08 |
| Sub042 | 3.32 | 1.43 | 0.31 | 0.47 | 0.55 | 24.16 | 17.73 | 550.00 | 0.01 | 0.00 | 0.02 | 0.01 | 0.01 | 0.08 |
| Ctr001 | 1.64 | 0.00 | 0.00 | 0.00 | 0.00 | 23.67 | 18.11 | 56.00 | 0.01 | 0.00 | 0.02 | 0.00 | 0.02 | 0.05 |
| Ctr002 | 2.94 | 0.98 | 0.00 | 0.00 | 0.00 | 24.86 | 17.12 | 95.00 | 0.01 | 0.00 | 0.00 | 0.00 | 0.02 | 0.06 |
| Ctr003 | 5.74 | 0.82 | 0.00 | 0.82 | 0.00 | 23.71 | 17.69 | 114.00 | 0.00 | 0.00 | 0.01 | 0.00 | 0.03 | 0.08 |
| Ctr004 | 3.53 | 1.18 | 0.00 | 0.00 | 0.00 | 24.69 | 17.85 | 84.00 | 0.00 | 0.01 | 0.03 | 0.00 | 0.01 | 0.07 |
| Ctr005 | 1.32 | 2.63 | 0.00 | 0.00 | 0.00 | 26.34 | 16.59 | 73.00 | 0.00 | 0.00 | 0.01 | 0.00 | 0.02 | 0.05 |
| Ctr006 | 2.68 | 0.00 | 0.00 | 0.00 | 0.00 | 24.68 | 19.60 | 106.00 | 0.00 | 0.00 | 0.01 | 0.00 | 0.03 | 0.05 |
| Ctr007 | 3.19 | 5.32 | 1.06 | 0.00 | 3.19 | 24.33 | 21.30 | 93.00 | 0.00 | 0.00 | 0.01 | 0.00 | 0.02 | 0.04 |
| Ctr008 | 2.11 | 1.05 | 0.00 | 1.05 | 0.00 | 22.98 | 19.52 | 93.00 | 0.01 | 0.00 | 0.03 | 0.00 | 0.01 | 0.08 |
| Ctr009 | 1.23 | 3.70 | 2.47 | 0.00 | 1.23 | 24.34 | 20.34 | 76.00 | 0.00 | 0.00 | 0.02 | 0.01 | 0.01 | 0.06 |
| Ctr010 | 4.35 | 1.74 | 0.00 | 0.00 | 0.87 | 25.85 | 16.78 | 121.00 | 0.01 | 0.00 | 0.02 | 0.01 | 0.02 | 0.07 |
| Ctr011 | 3.29 | 3.02 | 0.26 | 0.26 | 0.53 | 24.50 | 15.34 | 746.00 | 0.00 | 0.00 | 0.01 | 0.01 | 0.01 | 0.05 |
| Ctr012 | 1.05 | 5.79 | 0.00 | 0.53 | 4.74 | 24.11 | 14.29 | 189.00 | 0.00 | 0.00 | 0.02 | 0.01 | 0.01 | 0.06 |
| Ctr013 | 2.44 | 0.00 | 0.00 | 0.00 | 0.00 | 25.92 | 20.23 | 42.00 | 0.00 | 0.00 | 0.00 | 0.00 | 0.00 | 0.05 |
| Ctr014 | 5.68 | 1.14 | 1.14 | 1.14 | 0.00 | 23.57 | 18.66 | 85.00 | 0.01 | 0.00 | 0.01 | 0.01 | 0.01 | 0.11 |
| Ctr015 | 4.00 | 0.00 | 0.00 | 0.00 | 0.00 | 24.26 | 21.02 | 72.00 | 0.01 | 0.00 | 0.00 | 0.01 | 0.02 | 0.03 |
| Ctr016 | 10.53 | 0.00 | 0.00 | 0.00 | 0.00 | 25.29 | 20.92 | 53.00 | 0.00 | 0.00 | 0.01 | 0.00 | 0.00 | 0.03 |
| Ctr017 | 0.00 | 4.69 | 3.12 | 0.00 | 0.00 | 24.88 | 17.59 | 61.00 | 0.00 | 0.01 | 0.08 | 0.00 | 0.02 | 0.12 |
| Ctr018 | 3.05 | 2.29 | 0.00 | 0.00 | 2.29 | 23.14 | 19.94 | 132.00 | 0.00 | 0.01 | 0.01 | 0.00 | 0.00 | 0.06 |
| Ctr019 | 7.32 | 3.66 | 3.66 | 1.22 | 0.00 | 25.22 | 20.06 | 78.00 | 0.02 | 0.01 | 0.01 | 0.01 | 0.01 | 0.11 |
| Ctr020 | 2.15 | 1.08 | 1.08 | 0.00 | 0.00 | 24.54 | 14.21 | 85.00 | 0.01 | 0.00 | 0.03 | 0.01 | 0.01 | 0.03 |
| Ctr021 | 5.33 | 1.33 | 1.33 | 0.00 | 0.00 | 26.88 | 18.54 | 70.00 | 0.01 | 0.00 | 0.00 | 0.01 | 0.00 | 0.02 |
| Ctr022 | 6.67 | 0.00 | 0.00 | 0.00 | 0.00 | 22.71 | 18.71 | 57.00 | 0.05 | 0.01 | 0.01 | 0.00 | 0.00 | 0.05 |
| Ctr023 | 0.00 | 0.00 | 0.00 | 0.00 | 0.00 | 24.13 | 18.39 | 60.00 | 0.00 | 0.00 | 0.02 | 0.00 | 0.00 | 0.05 |
| Ctr024 | 15.07 | 0.00 | 0.00 | 0.00 | 0.00 | 24.11 | 20.43 | 73.00 | 0.01 | 0.00 | 0.00 | 0.01 | 0.02 | 0.12 |
| Ctr025 | 1.22 | 2.44 | 1.22 | 1.22 | 0.00 | 25.57 | 20.43 | 75.00 | 0.00 | 0.00 | 0.02 | 0.00 | 0.00 | 0.06 |
| Ctr026 | 4.00 | 4.00 | 3.00 | 0.00 | 0.00 | 24.45 | 18.03 | 95.00 | 0.01 | 0.00 | 0.01 | 0.00 | 0.01 | 0.07 |
| Ctr027 | 5.00 | 0.00 | 0.00 | 0.00 | 0.00 | 22.68 | 16.52 | 54.00 | 0.00 | 0.01 | 0.00 | 0.03 | 0.01 | 0.10 |
| Ctr028 | 4.60 | 0.00 | 0.00 | 0.00 | 0.00 | 22.94 | 19.73 | 81.00 | 0.00 | 0.00 | 0.05 | 0.04 | 0.00 | 0.07 |
| Ctr029 | 2.56 | 0.00 | 0.00 | 0.00 | 0.00 | 23.58 | 19.14 | 72.00 | 0.00 | 0.00 | 0.04 | 0.02 | 0.01 | 0.09 |
| Ctr030 | 5.06 | 0.00 | 0.00 | 0.00 | 0.00 | 25.07 | 19.65 | 75.00 | 0.00 | 0.00 | 0.00 | 0.00 | 0.01 | 0.07 |
| Ctr031 | 4.88 | 1.22 | 0.00 | 1.22 | 0.00 | 22.87 | 17.96 | 81.00 | 0.01 | 0.00 | 0.00 | 0.00 | 0.00 | 0.04 |
