# Supplementary material for: Dreaming during the Covid-19 pandemic: Computational assessment of dream reports reveals mental suffering related to fear of contagion
Source: PLoS One. 2020 Nov 30;15(11):e0242903. doi: 10.1371/journal.pone.0242903 (PMC7703999; doi:10.1371/journal.pone.0242903)
Supplement: S2 Table — PANSS psychometric scale is composed of 30 questions, graded 1 to 7 for each question, and subdivided into 3 sub-scales: positive symptoms, negative symptoms, and general symptoms. More details in Methods. (DOCX) [file pone.0242903.s002.docx]

**S2 Table:** Psychometric evaluations of participants during the pandemic period of observation. PANSS psychometric scale is composed of 30 questions, graded 1 to 7 for each question, and subdivided into 3 sub-scales: positive symptoms, negative symptoms, and general symptoms. More details in Methods.

| **ID** | **Psychometric Evaluation** | | | | **PANSS Negative Subscale** | | | | | | |
| --- | --- | --- | --- | --- | --- | --- | --- | --- | --- | --- | --- |
|  | **PANSS** | **Positive** | **Negative** | **General** | **N1** | **N2** | **N3** | **N4** | **N5** | **N6** | **N7** |
| Sub001 | 49 | 7 | 13 | 29 | 1 | 1 | 1 | 4 | 1 | 2 | 3 |
| Sub002 | 34 | 7 | 9 | 18 | 1 | 1 | 1 | 1 | 1 | 1 | 3 |
| Sub003 | 79 | 15 | 16 | 48 | 3 | 3 | 2 | 2 | 1 | 1 | 4 |
| Sub004 | 42 | 9 | 9 | 24 | 3 | 1 | 1 | 1 | 1 | 1 | 1 |
| Sub005 | 71 | 12 | 17 | 42 | 4 | 4 | 1 | 3 | 1 | 1 | 3 |
| Sub006 | 36 | 7 | 7 | 22 | 1 | 1 | 1 | 1 | 1 | 1 | 1 |
| Sub007 | 74 | 17 | 21 | 36 | 5 | 5 | 2 | 4 | 1 | 1 | 3 |
| Sub008 | 71 | 8 | 18 | 45 | 3 | 2 | 2 | 4 | 1 | 2 | 4 |
| Sub009 | 48 | 9 | 8 | 31 | 1 | 1 | 1 | 1 | 1 | 1 | 2 |
| Sub010 |  |  |  |  |  |  |  |  |  |  |  |
| Sub011 | 50 | 10 | 9 | 31 | 1 | 1 | 1 | 1 | 1 | 1 | 3 |
| Sub012 | 47 | 12 | 7 | 28 | 1 | 1 | 1 | 1 | 1 | 1 | 1 |
| Sub013 | 50 | 14 | 10 | 26 | 1 | 1 | 1 | 2 | 1 | 1 | 3 |
| Sub014 | 69 | 18 | 11 | 40 | 2 | 2 | 1 | 2 | 1 | 1 | 2 |
| Sub015 | 70 | 16 | 16 | 38 | 3 | 3 | 2 | 2 | 1 | 2 | 3 |
| Sub016 | 40 | 11 | 7 | 22 | 1 | 1 | 1 | 1 | 1 | 1 | 1 |
| Sub017 | 43 | 9 | 11 | 23 | 2 | 2 | 2 | 1 | 1 | 2 | 1 |
| Sub018 | 58 | 8 | 10 | 40 | 1 | 1 | 1 | 2 | 1 | 1 | 3 |
| Sub019 | 71 | 13 | 16 | 42 | 4 | 2 | 1 | 3 | 1 | 1 | 4 |
| Sub020 | 56 | 14 | 8 | 34 | 1 | 1 | 1 | 1 | 2 | 1 | 1 |
| Sub021 | 50 | 10 | 11 | 29 | 1 | 1 | 2 | 1 | 1 | 1 | 4 |
| Sub022 | 60 | 12 | 15 | 33 | 3 | 1 | 2 | 3 | 1 | 2 | 3 |
| Sub023 | 50 | 15 | 10 | 25 | 1 | 2 | 1 | 3 | 1 | 1 | 1 |
| Sub024 | 44 | 8 | 9 | 27 | 1 | 1 | 1 | 1 | 1 | 1 | 3 |
| Sub025 | 41 | 7 | 10 | 24 | 1 | 1 | 1 | 1 | 1 | 1 | 4 |
| Sub026 | 34 | 8 | 7 | 19 | 1 | 1 | 1 | 1 | 1 | 1 | 1 |
| Sub027 | 58 | 13 | 16 | 29 | 1 | 4 | 2 | 3 | 1 | 2 | 3 |
| Sub028 | 35 | 7 | 12 | 16 | 1 | 2 | 1 | 3 | 3 | 1 | 1 |
| Sub029 |  |  |  |  |  |  |  |  |  |  |  |
| Sub030 | 42 | 9 | 10 | 23 | 1 | 1 | 1 | 1 | 1 | 1 | 4 |
| Sub031 | 50 | 11 | 14 | 25 | 2 | 3 | 2 | 1 | 1 | 2 | 3 |
| Sub032 | 57 | 10 | 10 | 37 | 1 | 1 | 1 | 1 | 1 | 1 | 4 |
| Sub033 | 51 | 11 | 13 | 27 | 3 | 3 | 1 | 1 | 1 | 1 | 3 |
| Sub034 | 75 | 19 | 11 | 45 | 1 | 1 | 1 | 1 | 1 | 1 | 5 |
| Sub035 | 36 | 9 | 7 | 20 | 1 | 1 | 1 | 1 | 1 | 1 | 1 |
| Sub036 | 52 | 13 | 8 | 31 | 1 | 2 | 1 | 1 | 1 | 1 | 1 |
| Sub037 |  |  |  |  |  |  |  |  |  |  |  |
| Sub038 | 57 | 7 | 15 | 35 | 1 | 4 | 2 | 2 | 1 | 1 | 4 |
| Sub039 | 69 | 10 | 16 | 43 | 2 | 3 | 2 | 3 | 1 | 1 | 4 |
| Sub040 | 54 | 17 | 9 | 28 | 1 | 2 | 1 | 2 | 1 | 1 | 1 |
| Sub041 | 83 | 17 | 19 | 47 | 2 | 3 | 2 | 4 | 1 | 2 | 5 |
| Sub042 | 66 | 15 | 10 | 41 | 1 | 1 | 1 | 1 | 1 | 1 | 4 |
