# Supplementary material for: Dreaming during the Covid-19 pandemic: Computational assessment of dream reports reveals mental suffering related to fear of contagion
Source: PLoS One. 2020 Nov 30;15(11):e0242903. doi: 10.1371/journal.pone.0242903 (PMC7703999; doi:10.1371/journal.pone.0242903)
Supplement: S3 Table — The participants were asked to grade from 1 to 10 how they felt after the dream observation experience. Details in Methods. (DOCX) [file pone.0242903.s003.docx]

**S3 Table:** Self-evaluation of the dream observation experience during the first month of social isolation. The participants were asked to grade from 1 to 10 how they felt after the dream observation experience. Details in Methods.

| **ID** | **Dream Observation Self-evaluation Scale** | | | | | | | | | | | |
| --- | --- | --- | --- | --- | --- | --- | --- | --- | --- | --- | --- | --- |
|  | **Happy** | **Sad** | **Calm** | **Anxious** | **Energetic** | **Tired** | **Peaceful** | **Aggressive** | **Altruistic** | **Selfish** | **Creative** | **Confused** |
| Sub001 | 3 | 8 | 3 | 7 | 3 | 6 | 4 | 5 | 6 | 2 | 6 | 7 |
| Sub002 | 5 | 1 | 1 | 1 | 1 | 1 | 1 | 1 | 1 | 1 | 1 | 1 |
| Sub003 |  |  |  |  |  |  |  |  |  |  |  |  |
| Sub004 | 6 | 1 | 1 | 3 | 1 | 1 | 6 | 1 | 1 | 1 | 3 | 1 |
| Sub005 | 8 | 2 | 5 | 4 | 6 | 3 | 7 | 2 | 5 | 2 | 8 | 3 |
| Sub006 | 10 | 1 | 8 | 1 | 7 | 2 | 9 | 1 | 7 | 1 | 7 | 4 |
| Sub007 | 8 | 2 | 5 | 6 | 5 | 5 | 5 | 5 | 5 | 5 | 8 | 5 |
| Sub008 | 10 | 1 | 1 | 10 | 10 | 1 | 10 | 1 | 10 | 1 | 10 | 1 |
| Sub009 | 9 | 1 | 1 | 6 | 1 | 1 | 1 | 1 | 1 | 1 | 5 | 6 |
| Sub010 | 5 | 5 | 5 | 5 | 5 | 5 | 5 | 5 | 5 | 5 | 5 | 7 |
| Sub011 | 7 | 8 | 5 | 7 | 5 | 7 | 8 | 3 | 7 | 2 | 7 | 4 |
| Sub012 | 5 | 1 | 1 | 1 | 1 | 1 | 1 | 1 | 1 | 1 | 7 | 8 |
| Sub013 | 5 | 8 | 5 | 6 | 4 | 5 | 5 | 5 | 5 | 5 | 5 | 7 |
| Sub014 | 9 | 2 | 9 | 2 | 9 | 2 | 8 | 2 | 8 | 2 | 8 | 6 |
| Sub015 | 8 | 2 | 1 | 8 | 1 | 1 | 1 | 1 | 1 | 5 | 1 | 3 |
| Sub016 |  |  |  |  |  |  |  |  |  |  |  |  |
| Sub017 | 9 | 3 | 5 | 5 | 5 | 5 | 6 | 4 | 5 | 5 | 9 | 2 |
| Sub018 | 6 | 6 | 5 | 8 | 5 | 5 | 5 | 3 | 3 | 3 | 3 | 3 |
| Sub019 | 9 | 4 | 3 | 7 | 8 | 1 | 1 | 1 | 5 | 3 | 8 | 5 |
| Sub020 | 4 | 6 | 1 | 10 | 10 | 1 | 1 | 1 | 2 | 8 | 10 | 10 |
| Sub021 | 10 | 1 | 9 | 1 | 9 | 1 | 9 | 1 | 9 | 1 | 10 | 1 |
| Sub022 | 10 | 1 | 7 | 1 | 8 | 1 | 5 | 5 | 5 | 5 | 9 | 8 |
| Sub023 | 7 | 3 | 7 | 1 | 7 | 8 | 1 | 1 | 7 | 1 | 7 | 1 |
| Sub024 | 10 | 2 | 7 | 4 | 5 | 5 | 7 | 1 | 8 | 1 | 8 | 3 |
| Sub025 | 8 | 1 | 5 | 1 | 5 | 1 | 5 | 1 | 6 | 1 | 5 | 1 |
| Sub026 | 10 | 1 | 9 | 1 | 10 | 1 | 9 | 1 | 8 | 2 | 10 | 1 |
| Sub027 |  |  |  |  |  |  |  |  |  |  |  |  |
| Sub028 | 9 | 1 | 9 | 1 | 9 | 1 | 10 | 1 | 10 | 1 | 9 | 3 |
| Sub029 |  |  |  |  |  |  |  |  |  |  |  |  |
| Sub030 | 8 | 1 | 5 | 1 | 5 | 6 | 7 | 1 | 5 | 1 | 7 | 1 |
| Sub031 | 9 | 1 | 3 | 3 | 3 | 5 | 3 | 1 | 1 | 1 | 5 | 7 |
| Sub032 | 9 | 1 | 5 | 8 | 1 | 1 | 1 | 1 | 5 | 1 | 8 | 8 |
| Sub033 | 8 | 1 | 1 | 7 | 1 | 1 | 1 | 1 | 1 | 1 | 5 | 5 |
| Sub034 | 10 | 1 | 8 | 2 | 9 | 2 | 10 | 1 | 10 | 1 | 10 | 1 |
| Sub035 | 8 | 3 | 8 | 3 | 9 | 2 | 9 | 1 | 9 | 1 | 10 | 3 |
| Sub036 | 8 | 2 | 5 | 2 | 5 | 1 | 1 | 1 | 1 | 1 | 5 | 1 |
| Sub037 |  |  |  |  |  |  |  |  |  |  |  |  |
| Sub038 | 7 | 3 | 7 | 3 | 7 | 3 | 8 | 1 | 5 | 5 | 7 | 3 |
| Sub039 | 9 | 3 | 7 | 3 | 7 | 1 | 8 | 1 | 7 | 1 | 8 | 7 |
| Sub040 | 10 | 1 | 8 | 1 | 6 | 1 | 7 | 1 | 8 | 1 | 6 | 2 |
| Sub041 | 8 | 8 | 5 | 7 | 9 | 1 | 1 | 1 | 5 | 1 | 4 | 9 |
| Sub042 | 9 | 1 | 9 | 2 | 10 | 1 | 9 | 1 | 10 | 1 | 10 | 1 |
